# Bacterial Ortholog of Mammalian Translocator Protein (TSPO) with Virulence Regulating Activity

**DOI:** 10.1371/journal.pone.0006096

**Published:** 2009-06-30

**Authors:** Annelise Chapalain, Sylvie Chevalier, Nicole Orange, Laurence Murillo, Vassilios Papadopoulos, Marc G. J. Feuilloley

**Affiliations:** 1 Laboratory of Cold Microbiology UPRES EA4312, University of Rouen, Evreux, France; 2 ADIPpharm, Evreux, France; 3 The Research Institute of the McGill University Health Centre & Department of Medicine, McGill University, Montreal, Quebec, Canada; Baylor College of Medicine, United States of America

## Abstract

The translocator protein (TSPO), previously designated as peripheral-type benzodiazepine receptor, is a protein mainly located in the outer mitochondrial membrane of eukaryotic cells. TSPO is implicated in major physiological functions and functionally associated with other proteins such as the voltage-dependent anionic channel, also designated as mitochondrial porin. Surprisingly, a TSPO-related protein was identified in the photosynthetic bacterium *Rhodobacter sphaeroides* but it was initially considered as a relict of evolution. In the present study we cloned a *tspO* gene in *Pseudomonas fluorescens* MF37, a non-photosynthetic eubacterium and we used bioinformatics tools to identify TSPO in the genome of 97 other bacteria. *P. fluorescens* TSPO was recognized by antibodies against mouse protein and by PK 11195, an artificial ligand of mitochondrial TSPO. As in eukaryotes, bacterial TSPO appears functionally organized as a dimer and the apparent Kd for PK 11195 is in the same range than for its eukaryotic counterpart. When *P. fluorescens* MF37 was treated with PK 11195 (10^−5^ M) adhesion to living or artificial surfaces and biofilm formation activity were increased. Conversely, the apoptotic potential of bacteria on eukaryotic cells was significantly reduced. This effect of PK11195 was abolished in a mutant of *P. fluorescens* MF37 deficient for its major outer membrane porin, OprF. The present results demonstrate the existence of a bacterial TSPO that shares common structural and functional characteristics with its mammalian counterpart. This protein, apparently involved in adhesion and virulence, reveals the existence of a possible new inter kingdom signalling system and suggests that the human microbiome should be involuntarily exposed to the evolutionary pressure of benzodiazepines and related molecules. This discovery also represents a promising opportunity for the development of alternative antibacterial strategies.

## Introduction

Benzodiazepines are among the most widely prescribed drugs in the world [1] and are known as sedative-hypnotic agents efficient against anxiety, sleep disorders and related troubles. The first identified target of benzodiazepines, designated as central benzodiazepine receptor, is corresponding to a secondary site of subtype A γ-aminobutyric acid receptors (GABA_A_ receptors) expressed in neurons. However, a second type of benzodiazepine receptor was identified in mammals in 1977. This binding site was named peripheral-type benzodiazepine receptor (PBR) [2]. As the structure of this receptor is complex and variable there was rapidly a large confusion, the same word being used to designate the multimeric assembling or the benzodiazepine binding protein itself. For that reason, on the basis of its identified functions in eukaryotes it was proposed to designate this benzodiazepine binding protein as “Translocator Protein” (TSPO) [2].

For a receptor, the sub-cellular localization of TSPO is original since it is essentially present in the outer mitochondrial membrane [3]. As it has also been found in nuclear and cytoplasmic membranes it was proposed to distinguish mitochondrial TSPO and nuclear TSPO [2]. In the mitochondrial membrane, TSPO is functionally associated with a Voltage Dependant Anionic Channel (VDAC), also designated as mitochondrial porin, and with an Adenine Nucleotide Translocase (ANT) essentially associated with inner membrane [4]. TSPO is present in all mammalian tissues with the exception of neurons, but differently expressed according to the cellular function [2], [5]. TSPO has a major role in steroidogenesis and is also implicated in porphyrin biosynthesis, cell proliferation and apoptosis [5]. Natural ligands of TSPO are cholesterol, Diazepam Binding Inhibitor (DBI) or protoporphyrin IX; principal artificial ligands are isoquinoline carboxamide, such as PK 11195, and benzodiazepines, such as Ro5-4864. However, benzodiazepine binding requires the presence of VDAC, whereas PK 11195 binding does not require the presence of other proteins [5].

TSPO was mostly studied in mammals but this protein is also expressed in invertebrates [6] and vegetal [7]. Surprisingly, a functional homologue of TSPO has been identified in the photosynthetic bacterium, *Rhodobacter sphaeroides* [8], [9]. In this prokaryote, TSPO negatively regulates pigment biosynthesis and photosynthesis in response to light and oxygen modifications [8]. TSPO-related proteins have been identified later in the cyanobacterium, *Fremyella diplosiphon* [10] and in the non-photosynthetic bacterium, *Sinorhizobium meliloti* [11]. *R. sphaeroides* and *S. meliloti* are both members of the alpha subdivision of purple bacteria, the organisms that likely gave rise to the endosymbiont at the origin of mitochondria. Therefore, the existence of TSPO in these micro-organisms was considered to be a relict of evolution.

Here we show that a functional ortholog of *tspO*, *i.e.* a gene that has evolved from a common ancestor, is expressed in *Pseudomonas fluorescens,* a ubiquitous micro-organism of the gamma-proteobacteria subdivision of eubacteria. The distribution of homologous genes in different bacterial species or strains covering most taxonomic groups was established. In addition to structural homology to mitochondrial TSPO, *P. fluorescens* TSPO has common pharmacological properties, as demonstrated by its high affinity for PK 11195. The use of PK 11195 revealed that this bacterial TSPO is involved in the regulation of adhesion and virulence and, as a mitochondrial TSPO should be, functionally associated with a membrane porin. These results suggest that natural ligands of TSPO, that remain to be identified, should be involved in inter-kingdom communication. Moreover, it appears that the behavior of bacteria in the human microbiome can be modulated by current pharmaceutical agents like benzodiazepines. On another side, these results suggest a new strategy for the development of bacterial inhibitors of virulence usable in complement or in place of antibiotics.

## Results

### A *tspO* gene is present in the γ-proteobacterium *Pseudomonas fluorescens*

**Figure 1:**
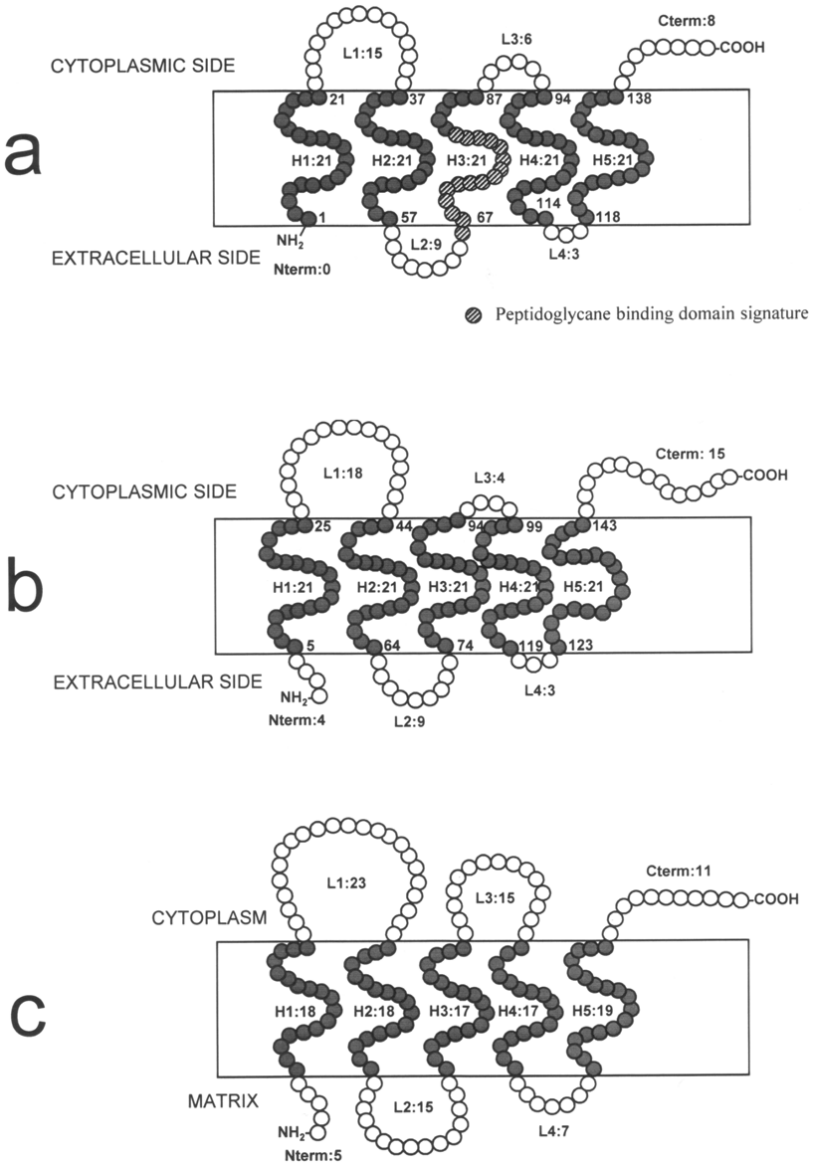
Comparison of the putative topological organization of bacterial and mitochondrial TSPO. (a) *Pseudomonas fluorescens* MF37 bactTSPO. (b) BactTSPO from *Rhodobacter sphaeroides* 2.4.1 recalculated from Yeliseev and Kaplan [12]. (c) Mitochondrial TSPO [13]. *P. fluorescens* MF37 bactTSPO has a typical five transmembrane helix structure, a larger L1 intra-cytoplasmic loop, and an extended cytoplasmic C-terminal end. In contrast, the extracellular N-terminal end is absent, and a peptidoglycane binding domain signature is present between the L2 loop and H3 transmembrane domain.

**Figure 2:**
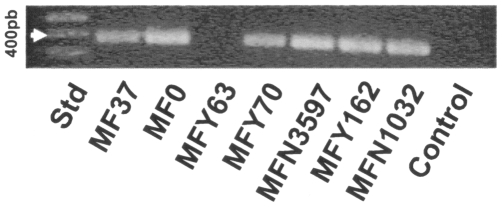
Identification of *tspO* genes in fluorescent *Pseudomonas*. PCR amplification of *tspO*-encoding sequences in the biovar V strains *Pseudomonas fluorescens* MF37 and MF0, the clinical biovar II strain *Pseudomonas fluorescens* MFY70, the biovar B strain *Pseudomonas putida* MFN3597, and the biovar I strains, *Pseudomonas fluorescens* MFY162 and MFN1032. The biovar II strain, *Pseudomonas fluorescens* MFY63, lacks *tspO*-related sequences. Std: Molecular mass standards.

In order to investigate the presence of a *tspO* gene in non-photosynthetic and typical eubacterium, the published genome of the environmental and widespread micro-organism *Pseudomonas fluorescens* (strain SBW25) was screened by BlastP against the TSPO sequence of *R. sphaeroides* 2.4.1. A sequence encoding a protein with 50% identity to 2.4.1 TSPO of *R. sphaeroides* was detected *in silico* in the genomic sequence of *P. fluorescens* SBW25. In order to verify experimentally the presence of this gene in *P. fluorescens*, primers were designed from the *P. fluorescens* SBW25 *tspO* gene sequence and used in our reference strain, *P. fluorescens* MF37. A PCR-amplified fragment of 438 bp, encoding 146 amino acids was produced from *P. fluorescens* MF37 (NCBI accession number FJ185105). The fragment was cloned into the pMOSBlue plasmid and sequenced. A BlastP search of the full fragment sequence confirmed that it was a TSPO-like protein. Calculation of the topological structure of this protein by TopPred revealed a five transmembrane helix structure analogous to that of *R. sphaeroides* 2.4.1 [12] and mitochondrial TSPO [13], though the extracellular N-terminus is absent and the C-terminus shortened (**Fig. 1**). The first cytoplasmic loop is highly conserved (80% similarity including 46.7% identity) between *R. sphaeroides* 2.4.1 and *P. fluorescens* MF37. Moreover, Lys21 and Lys31 (corresponding to residues 26 and 36 in *R. sphaeroides* 2.4.1.) are fully conserved in both amino acid identity and the distance between the two residues. The software PSORTb indicated with high probability association of TSPO with the inner bacterial membrane and it was interesting to note that it was not possible to extract TSPO from the sub-fraction corresponding to the outer membrane of the bacterium. In agreement with its probable membrane localization, a peptidoglycane-binding domain signature was detected between residues 66 and 81 in the third transmembrane helix. Using specific primers designed from the *P. fluorescens* MF37 *tspO* gene sequence, we successfully amplified homologous sequences in other strains of *P. fluorescens*, including the biovar V strain, MF0, as well as the clinical biovar I and II strains, MFY70, MFY162, and MFN1032 (**Fig. 2**). Intriguingly, a *tspO* fragment could be amplified from the MFN3597 strain, previously identified as *P. putida* [14], but not from the clinical strain, *P. fluorescens* MFY63 (biovar II). Sequencing and alignment of the translated TSPO sequences from the amplified fragments revealed 90% similarity. An unexpected result was the impossibility to amplify a *tspO* gene in *Pseudomonas aeruginosa* PAO1 (data not shown).

### *TspO* orthologs are widely, but heterogeneously distributed in eubacteria

**Figure 3:**
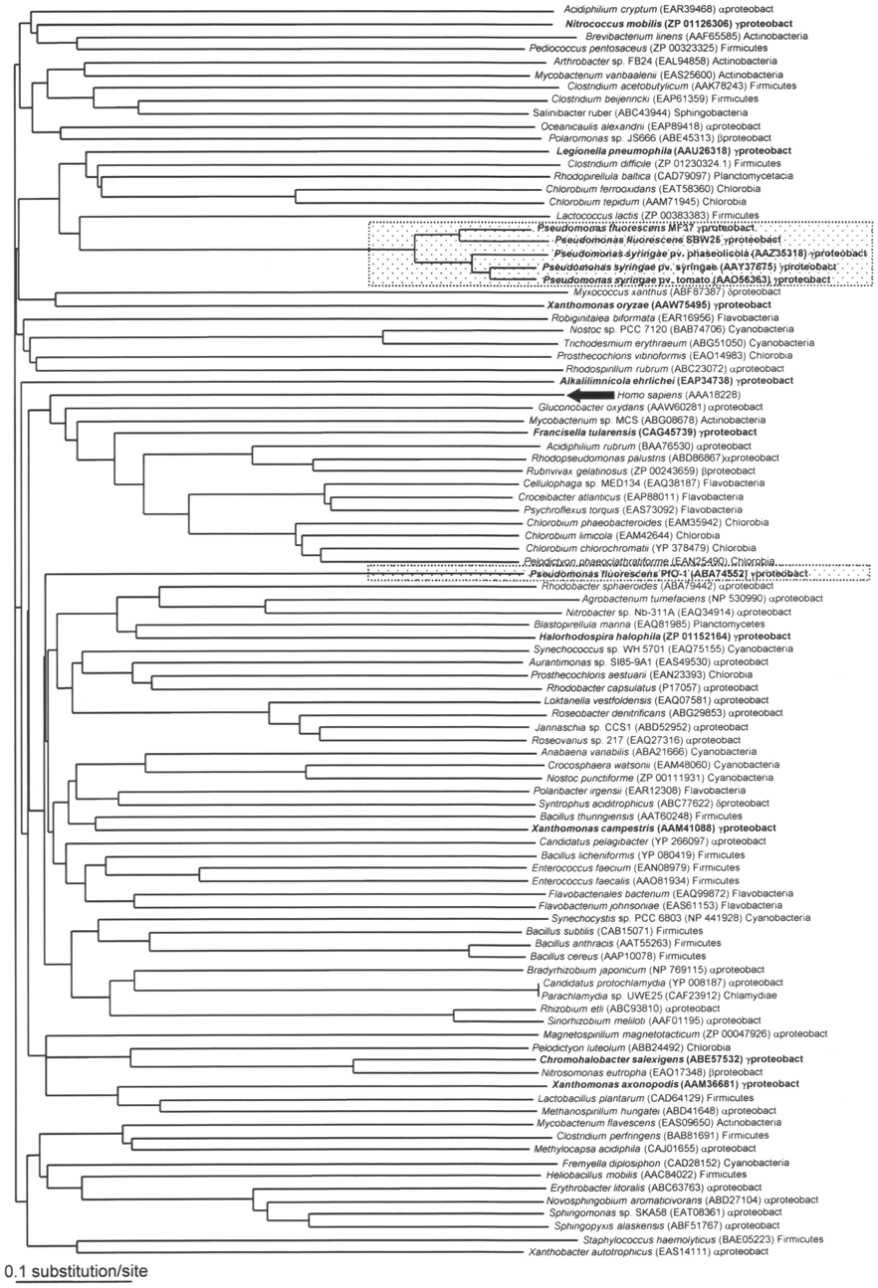
Amino acid sequence cladogram showing the relationships between bacterial TSPO. Bacterial names, accession numbers, and taxonomic groups are indicated for each branch. Bold characters indicate γ-proteobacteria, and the black arrow indicates the position of TSPO expressed in human mitochondria. The two dotted boxes indicate fluorescent *Pseudomonas* strains containing a bactTSPO gene (*P. fluorescens* and *P. syringae*). The degree of statistical support for branches was determined with 1000 bootstrap replicates. All branches showed less than 30% divergence.

**Figure 4:**
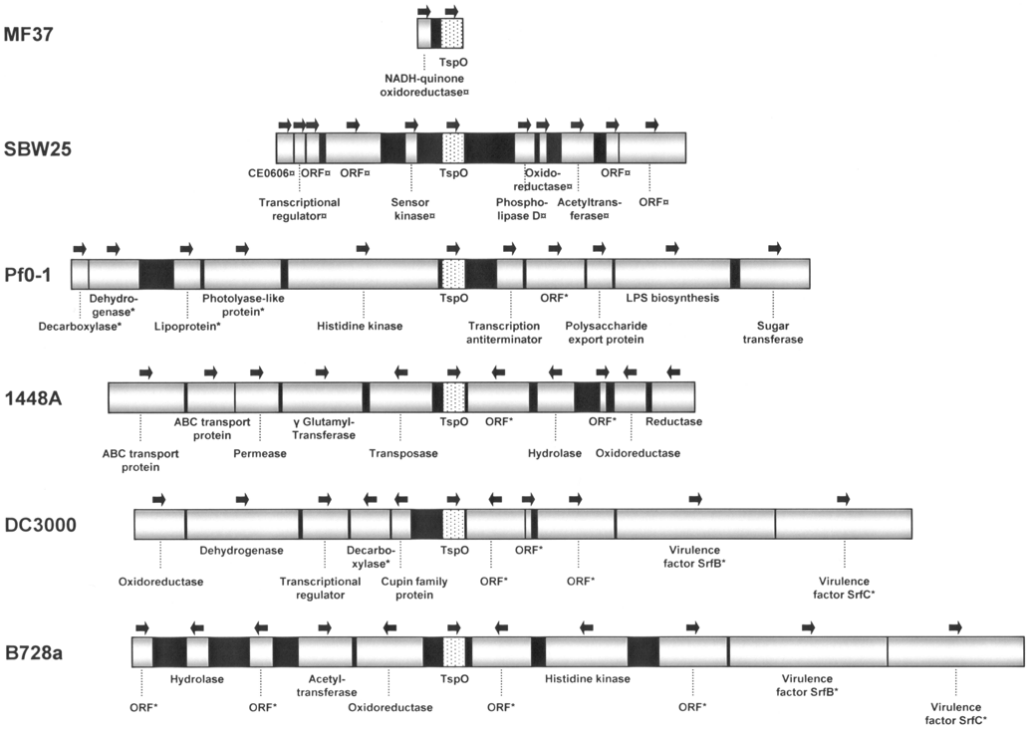
Comparison of *tspO* genomic environments among *Pseudomonas*. Physical maps of the regions upstream and downstream of *tspO* are shown for *P. fluorescens* strains MF37, SBW25, and Pf0-1 as well as *P. syringae* strains 1448A (pathovar phaseolicola), DC3000 (pv. tomato), and B728a (pv. syringae). ORF: non-identified putative protein, ¤ Data obtained from Blast, * Protein putative function. Black areas represent intergenic regions.

Since our results suggested a broader distribution of TSPO than expected, the TSPO sequence from *P. fluorescens* MF37 was used to search for homologous proteins in sequenced bacterial genomes available in the NCBI databank. We found that 97 different eubacterial species or strains encompassing most of the different taxonomic groups contained a TSPO-related protein that we propose to designate as bacterial TSPO. Bacterial TSPO appears to be widely distributed in eubacteria, including human pathogens such as *Bacillus anthracis*, *Legionella pneumophila*, *Staphylococcus haemolyticus*, and *Clostridium perfringens*. A cladogram was constructed based on the 97 bacterial TSPO sequences (**Fig. 3**). Although the sequence of human mitochondrial TSPO was included in the series, the tree remained unrooted. In addition, it was not possible to distinguish any specific clusters grouping homogenous bacteria, with the single exception of a cluster of fluorescent *Pseudomonas* that included *P. fluorescens* MF37 and SBW25, as well as the *P. syringae* pathovars *phaseolicola*, *syringae*, and *tomato*. In fact, this cluster was certainly an artifact due to the presence of several identified sequences from closely related bacteria. Even the *P. fluorescens* Pf0-1 strain remained separate from the cluster of other fluorescent *Pseudomonas*. The absence of *tspO* in *P. aeruginosa* was confirmed by BlastP search in the strain PAO1 and in all genomic sequences available on the site *Pseudomonas.com*, *i.e.* strains PA7, PA14 and LESB58. In contrast, homologous sequences were found in different archaeal prokaryotes (data not shown). The genomic environment of *tspO* in fluorescent *Pseudomonas* was analyzed. The sequences flanking *tspO* in *P. fluorescens* MF37 were sequenced and those in *P. fluorescens* Pf0-1, SBW25, *P. syringae pv. phaseolicola* 1448A, *pv. syringae* B728a, and *pv. tomato* DC3000 were retrieved from the NCBI databank. The sense, size, and putative functions of the genes in the flanking regions of *tspO* were different in the six bacterial types but we observed that the *tspO* environment was particularly rich in transposase genes (**Fig. 4**). In *P. syringae pv. phaseolicola*, a gene encoding a transposase lies immediately upstream of *tspO* (position 1) with four additional transposase genes lying upstream (position 14) or downstream (positions 15, 16 and 17) of *tspO*. Sequences encoding transposases were also observed near *tspO* in another two strains of *P. syringae* (DC3000 strain: upstream gene positions 16 and 17 and downstream positions 23, 24, 25, 26, and 29; B728a strain: upstream position 31 and downstream position 17 and 31) and in *P. fluorescens* Pf0-1 (upstream position 17). The GC content of the upstream and downstream sequences was determined in the different *Pseudomonas* whose genomic environment was studied. Differences between the GC contents of the *tspO* gene in a same bacterial species were generally lower than 3% (maximum 5%) whereas inter-species variations reached 9%.

### *TspO* is differently expressed in fluorescent *Pseudomonas*

**Figure 5:**
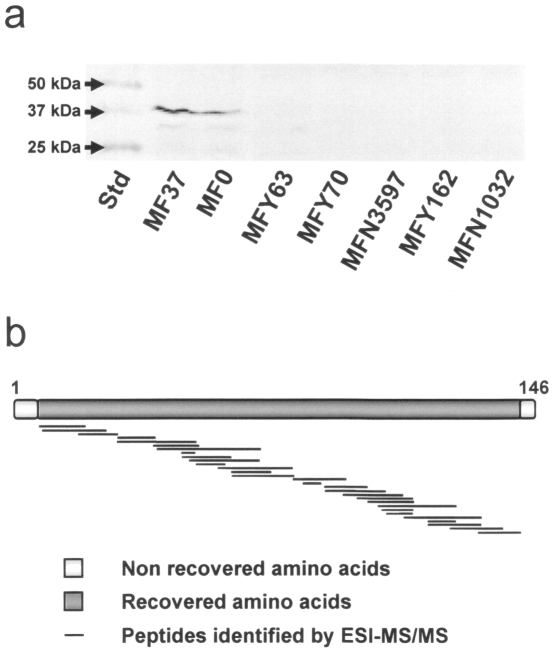
Expression of TSPO in fluorescent *Pseudomonas*. (a) Western blot analysis of total proteins extracts from the biovars V *Pseudomonas fluorescens* MF37 and MF0, the clinical strains of *Pseudomonas fluorescens* MFY70, MFY162, and MFN1032, and *Pseudomonas putida* MFN3597. (b) Alignment of the fragments obtained by ESI-MS/MS analysis of the TSPO immunoreactive bands extracted from MF37 and showing 92% recovery with the theoretical sequence of the protein. Std: Molecular mass standards.

As the presence of a gene is not a warranty of its functionality, expression of the *tspO* gene was studied in *P. fluorescens* MF37 and related strains by RT-PCR and western blot analysis of total protein extracts using an antibody raised against mouse TSPO. RT-PCR revealed the expression of mRNA coding TSPO in extracts from *P. fluorescens* MF37 grown in normal or in reduced oxygen conditions without visible difference (data not shown). Western blot analysis of *P. fluorescens* MF37 extracts allowed to detect a TSPO immunoreactive band corresponding to a 37 kDa protein (**Fig. 5a**). This mass was double what was theoretically expected for bacterial TSPO (17 kDa) but ESI-MS/MS analysis (**Fig. 5b**) indicated a 92% recovery between experimental and theoretical masses and confirmed that this immunoreactive band was actually bacterial TSPO. A same protein was detected in extracts of *P. fluorescens* MF0, the strain from which MF37 was produced [15]. In contrast, although PCR allowed us to amplify TSPO homologous sequences in *P. fluorescens* MFY70, MFY162, MFN1032, and in *P. putida* MFN3597, western blot analysis did not detect the expression of bacterial TSPO-like immunoreactive proteins in these micro-organisms.

### Bacterial TSPO expressed in *Pseudomonas fluorescens* MF37 shares pharmacological properties with eukaryotic mitochondrial TSPO

**Figure 6:**
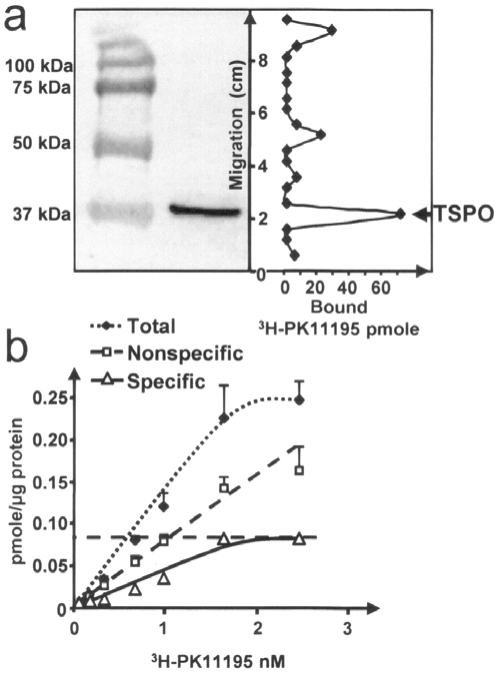
Binding of PK 11195 to bacterial TSPO of *Pseudomonas fluorescens* MF37. (a) Demonstration of the binding of [^3^H] PK 11195 at the position of the TSPO-immunoreactive band visualized by western blots in *P. fluorescens* MF37 extracts. (b) Scatchard plot showing the binding characteristics of PK 11195 to TSPO of *P. fluorescens* MF37. The maximal binding potential (Bmax) is 0.087 pmole.µ g^−1^ bacterial protein and the Kd is 0.92 nM.

The pharmacological properties of *P. fluorescens* MF37 bacterial TSPO were investigated using a specific ligand of eukaryotic mitochondrial TSPO, PK 11195 [16]. A total protein extract of MF37 was separated by SDS-page electrophoresis, transferred onto nitrocellulose membrane, and hybridized with tritiated PK11195 ([^3^H]-PK 11195). The main binding peak of [^3^H]-PK 11195 was detected at the position of the 37 kDa band identified by western blot as containing MF37 bacterial TSPO (**Fig. 6a**). Secondary binding peaks were observed on proteins of higher molecular mass that were not recognized by the TSPO antiserum. A Scatchard study was then performed using the same tracer and membranes purified from *P. fluorescens* MF37. The Kd determined under these conditions (0.92 nM) in the same range as what has been previously established for PK 11195 binding to mammalian mitochondrial TSPO (1.5 nM) [17] (**Fig. 6b**). These results suggest that *P. fluorescens* MF37 bacterial TSPO recognizes PK 11195 as a ligand, and we decided to use this molecule to investigate the functional role of MF37 TSPO.

### Bacterial TSPO regulates the adhesion and cytotoxic activity of *Pseudomonas fluorescens*

**Figure 7:**
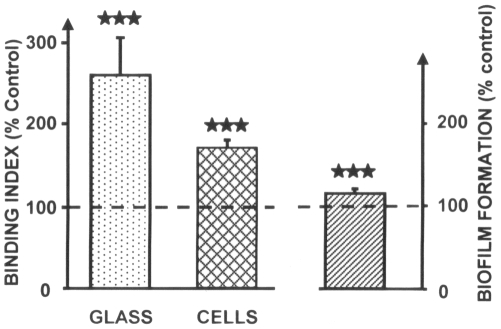
Effect of PK 11195 on *Pseudomonas fluorescens* MF37 adhesion to glass or eukaryotic cells, and on biofilm formation on PVC. Results are expressed as percentages of the values measured in the absence of treatment (100%). These changes were not associated with variations in the global surface polarity of bacteria. ***P<0.001.

**Figure 8:**
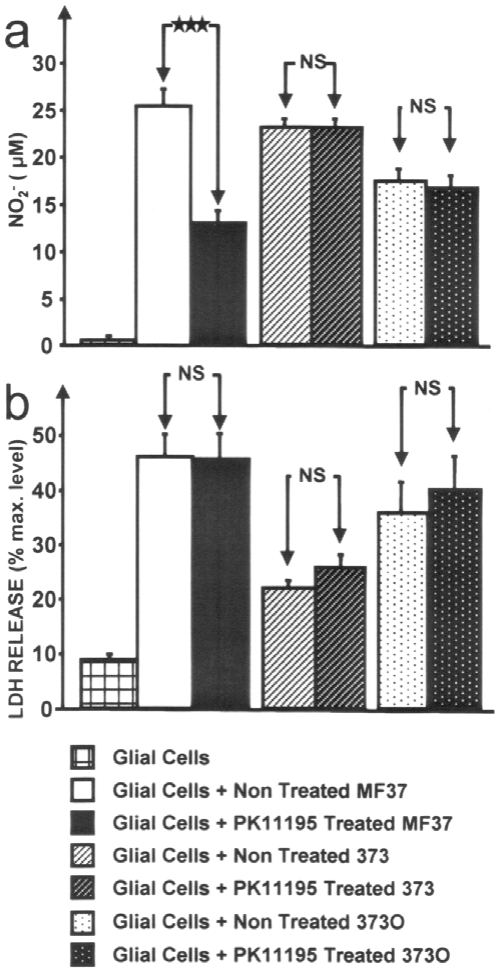
Effect of PK 11195 on the cytotoxicity of *Pseudomonas fluorescens* MF37 and its *OprF*-deficient (373) and complemented *OprF*-deficient (373O) mutants. (a) Effect of PK 11195 (10^−5^ M) on the apoptotic-like activity of the bacteria determined by measuring nitrite production (NO_2_^−^) resulting from NO biosynthesis by eukaryotic cells. (b) Effect of PK 11195 (10^−5^ M) on necrotic-like effect of *P. fluorescens* MF37, 373 and 373O determined by lactate dehydrogenase (LDH) activity released by eukaryotic cells. ***P<0.001. NS: not significant.

Owing to the role of TSPO in the adaptation of *R. sphaeroides* to changes in oxygen concentrations [9], we first investigated the effect of PK 11195 on the growth kinetics of *P. fluorescens* MF37 cultured under normal aerobic or low oxygen conditions. Exposure of the bacteria to PK 11195 (10^−5^ M) at any time of the growth phase (*i.e*., at the onset of growth, mid-exponential growth phase, or at the beginning of stationary phase) did not alter the growth kinetics. Since, in *Pseudomonas*, mobility is indirectly controlled by oxygen concentration [18], we investigated the effect of PK11195 on bacterial mobility and adherence potential. Treatment with PK 11195 did not alter the swimming or swarming mobilities of *P. fluorescens* MF37. In contrast, the adhesion potential of bacteria on glass and on eukaryotic (glial) cells was markedly increased (+168±69% and +70±9%, respectively) by PK 11195 exposure (**Fig. 7**). The ability of *P. fluorescens* MF37 to form biofilms on polystyrene was also significantly increased, albeit to a limited degree (+16±4%). However, these changes in the adhesion potential of *P. fluorescens* were not associated with global variations in surface polarity, as demonstrated by measuring the bacterial affinity to solvent. *P. fluorescens* MF37 is non-invasive on glial cells and PK 11195 does not modify this behavior (data not shown). Given that bacterial adhesion and virulence are linked, we investigated the effect of PK 11195 on the cytotoxicity of *P. fluorescens* MF37 using the release of nitrites and lactate dehydrogenase (LDH) as markers of apoptosis and necrosis, respectively [19]. In these experiments, bacteria were grown with PK 11195 (10^−5^ M, 24 h) and carefully rinsed to remove any trace of ligand before transferring them to the growth medium of eukaryotic (glial) cells. Under these conditions, the apoptotic-like effect of *P. fluorescens* MF37 was strongly decreased (−49±18%) (**Fig. 8**). Similar results were observed at doses of PK 11195 ranging from 10^−6^ to 10^−8^ M. The same experiment was repeated using *P. fluorescens* 373, a mutant of MF37 lacking the gene for OprF (the major porin of the bacterium) and *P. fluorescens* 373O, the *oprF*-complemented mutant. The apoptotic-like effect of the mutant 373 was identical to that of MF37 but its necrotic-like activity was significantly reduced. PK 11195 had no effect on the cytotoxicity of this *oprF* deleted mutant of *P. fluorescens*. *OprF*-transcomplementation allowed partial reversion of the phenotype with almost total restoration of the original necrotic-like activity of bacterium. However to the apoptotic-like activity was reduced in the *OprF*-complemented mutant 373O and it was not possible to visualize restoration of the sensitivity to PK 11195.

## Discussion

The results of the present study demonstrate the existence of a *tspO* gene in a wide variety of bacterial species and its expression as bacterial TSPO protein in the micro-organism *Pseudomonas fluorescens*, where its role in virulence regulation appears to be entirely different than initially proposed for *Rhodobacter sphaeroides* and α-proteobacteria. As observed in the case of other proteins [20], [21] whose structures were strongly preserved during evolution, functions can evolve independently and the present results reveal that it is necessary to reconsider the way we interpret the presence of TSPO in bacteria.

*Pseudomonas*, and particularly *Pseudomonas fluorescens*, are ubiquitous and non-photosynthetic micro-organisms that diverged long time ago from the ancestor of *Rhodobacter sphaeroides*. Because of the high versatility of this micro-organism [22] we conducted most of our bioinformatic studies on the basis of the bacterial TSPO sequence from the reference strain, *P. fluorescens* MF37. *P. fluorescens* MF37 is a typical psychrotrophic bacterium member of the biovar V subdivision of the species and its physiological characteristics have been extensively investigated in our laboratory. In addition to cloning of the *tspO* gene of *P. fluorescens* MF37, we attempted to produce a deletion mutant by different techniques ranging from simple crossing over and cloning in pMOS, pME3087 or pKNOCK suicide vectors to double crossing over using the Cre-lox system but we never succeeded. In eukaryotes deletion of the tspO gene is lethal [23] suggesting involvement of TSPO in essential physiological functions and our observations indicate that it should be also the same in *P. fluorescens*. We propose to designate this protein as “bacterial TSPO” in accordance with the names of “mitochondrial TSPO” and “nuclear TSPO” in eukaryotes [2].

The topological structure deduced from the TSPO sequence of *P. fluorescens* MF37, characterized by a five transmembrane helix, is analogous to that of *R. sphaeroides* 2.4.1 and mitochondrial TSPO [12], [13], though it appears to be partially or completely truncated at the N- and C-terminus. The structure of the TSPO molecule is particularly well preserved in the regions essential for its function, such as the first cytoplasmic loop, considered to be the main ligand-binding domain of the protein [12]. However, there is a divergence of orientation between bacterial and mitochondrial TSPO. In mitochondria, the N-terminus of TSPO is oriented in direction of the matrix [13]. In contrast, in *R. sphaeroides* bacterial TSPO appears inserted in the opposite sense, *i.e.* with the N-terminus exposed to the extracellular side [12]. A same orientation was predicted by TopPred for TSPO in *P. fluorescens*. The origin of this inversion of orientation should be ascribed to the fact that in eukaryotes TSPO is encoded in the nuclear genome and translocated to the outer membrane of the mitochondrium [24] whereas in bacteria, which synthesise TSPO in their cytoplasm, the protein has to be inserted from the inside of the prokaryotic cell. The localization of TSPO in the membranes of *P. fluorescens* is apparently different from that previously found. In *R. sphaeroides* [12], as in mitochondria [2], TSPO is shown associated to the outer membrane. In contrast, calculations using the software PSORTb and extraction tests on bacterial sub-membrane fractions both suggest association of TSPO to the inner membrane of *P. fluorescens*. In support of this hypothesis it is known that in Gram-negative bacteria, proteins folded in α-helixes are essentially, if not exclusively, associated to the inner membrane [25]. The presence of a peptidoglycan binding domain in the C terminal region of *P. fluorescens* TSPO is consistent with association of this protein to the bacterial membranes. A similar peptidoglycan binding signature was also observed in the porin OprF of *Pseudomonas aeruginosa* [26] and OmpA of *Erwinia carotovora* [27] but the role of this hidden domain in TSPO is unexplained. Experimental studies could reveal differences with predictive model, as described for mammalian TSPO by Murail *et al.* [28] making possible that the peptidoglycan domain of TSPO was more exposed to the surface and have same function of anchoring to periplasmic peptidoglycan as described for the C terminal region of *P. aeruginosa* OprF [26].

Since we have collected strains of *P. fluorescens* from diverse environments, including from clinical origins, where they were identified as opportunistic pathogens [14], we searched for the presence of a tspO gene in various biovar and temperature variants of the species. Primers derived from the MF37 tspO gene sequence revealed that homologous sequences are indeed present, not only in the genomes of biovar I, II, and V of the species, but also in a closely related species, Pseudomonas putida. The high degree of similarity of the translated sequences (90%) suggests that the structure of TSPO is conserved between these bacteria. Therefore, we were particularly surprised by our failure to detect a tspO-like sequence in one clinical strain of *P. fluorescens* and in Pseudomonas aeruginosa PAO1. As confirmed later in an in-silico study on all eubacterial sequences available in the NCBI databank, the distribution of the tspO gene is wide but very irregular. In addition, although we could build a cladogram over 97 bacterial TSPO sequences, it was impossible to define specific clusters corresponding to sub-types of this protein. The irregular presence of TSPO in eubacteria can not be simply explained by a lack of homogeneity of the species whose genomes were registered in the NCBI data-bank or by an insufficient volume of data. In contrast, as suggested by the detailed study of the genomic environment of tspO in fluorescent Pseudomonas, this situation probably reflects an original mechanism of acquisition of TSPO. Indeed, in the different genomes of Pseudomonas where TSPO was detected, we noted at the vicinity of the tspO gene the presence of genes encoding transposases. This topographical organization should favor gene exchange and, consequently, the irregular distribution of the protein. Although, there is no precise limit values for differences between CG contents in individual genes, the low level of variations of the GC contents between tspO and the neighboring genes in each individual species or strains [29] suggests that tspO does not results from a recent gene transfer. In addition, as indicated from our western blot studies, TSPO expression is precisely regulated. Whereas a tspO gene was detected in 5 of the 6 strains of *P. fluorescens* studied and alignment of the translated sequences suggested a high degree of homology of the protein, the TSPO protein was only detected in MF37 and in its parental strain, MF0. The identity of the protein was confirmed by ESI-MS/MS analysis, although the apparent mass of the protein was double what was predicted, suggesting that, as shown by Yeliseev & Kaplan [12] and Delavoie et al. [30], bacterial TSPO exists as a dimer. Secondary binding peaks for the specific ligand of TSPO PK 11195 were observed but they should not represent trimers or tetramers of TSPO since they were not recognized in western blot by the antiserum. The disparities between the results of PCR and western blot analysis could result either from conformational variations making TSPO undetectable by the antiserum (an unlikely situation because the polyclonal antiserum shows cross reactivity with different forms of TSPO, including those of vegetal origin [7]), or from the existence of specific regulatory mechanisms in the different strains which could repress TSPO expression in our experimental conditions. Altogether, these results suggest that bacterial TSPO is a protein whose acquisition and expression is precisely regulated, as is normally observed for molecules with important physiological functions.

In the bacterial TSPO of MF37, the high degree of conservation in the region considered to be the ligand-binding domain of the protein is consistent with our observation that PK 11195, a high-affinity ligand of mitochondrial TSPO, was also recognized by this protein with nanomolar affinity. This observation provided an opportunity to directly probe the function of bacterial TSPO in *P. fluorescens*. PK 11195 did not modulate bacterial growth and mobility, and this result was independent of the oxygenation conditions of the bacterium. This is different from what has been observed in *R. sphaeroides*, where TSPO is considered to be an oxygen sensor [9], and is not surprising considering *P. fluorescens* is not a photosynthetic bacterium and that the metabolism, structural, and even genomic organization of the two micro-organisms have very few common points. The observation that PK 11195 increases the adhesion of *P. fluorescens* on diverse surfaces ranging from glass to eukaryotic cells and also stimulates the biofilm formation potential of bacteria on polystyrene is essential, since bacterial adhesion is considered a key point in virulence expression [31]. Bacterial adhesion is mediated by at least three types of forces generated by surface polarity, pili and flagella, or specific membrane proteins. As PK 11195 has the same effect on bacterial adhesion to polar (glass), complex (eukaryotic cells), and hydrophobic surfaces (polystyrene) and does not modify the affinity to solvents of the bacteria, global variations of surface polarity can be excluded. Considering that the flagellar-dependent mobility of *P. fluorescens* was not influenced by PK 11195 treatment, our results suggest that this molecule acts on the configuration and/or activity of a specific membrane adhesin. In *P. fluorescens*, the porin OprF is the major outer membrane protein and implication of this molecule in adhesion to living cells [32], matrix protein [33], and abiotic surfaces [34] has already been demonstrated. As we did not observe any binding of PK 11195 to OprF by western blot, we can only postulate an indirect action of the drug on this protein, a hypothesis that was tested in the last part of the study.

When *P. fluorescens* MF37 was pre-treated with PK 11195, its apoptotic-like effect on eukaryotic cells (glial cells) was significantly reduced. In contrast, the necrotic-like effect of the bacterium was not changed, suggesting that PK 11195 was not acting on the release of enzymes and/or soluble toxins, but was modulating a specific apoptotic signal generated by the bacterium, and probably associated with outer membrane surface components. The same experiment was conducted in a *P. fluorescens* OprF-deficient mutant derived from MF37 (MF373). The apoptotic-like effect of MF373 was identical to that of its parent strain whereas its necrotic activity was markedly reduced. This difference should be ascribed to the absence of OprF which is an adhesin required for contact mediated cytotoxicity [32], [33] and has intrinsic cytotoxic potential [35], [36]. In this *oprF*-deleted strain, PK 11195 does not modules the apoptotic-like activity of the bacterium. These results indicate that PK 11195 acts on a process that regulates the virulence of *P. fluorescens* and requires expression of OprF. The necrotic-like activity of *P. fluorescens* was almost completely restored in the *oprF*-transcomplemented strain *P. fluorescens* 373O whereas its apoptotic effect was reduced. In these conditions it was not possible to observe a restoration of the inhibitory activity of PK 11195 on apoptosis. This partial phenotypic reversion in the complemented strain can be explained by the fact that plasmidic *oprF*-transcomplementation does not fully reproduces the regulation exerted over the endogenous chromosome-bound gene and underlines the phenotypic sensitivity of *Pseudomonas* to plasmidic of chromosomal transformations.

From these results, it is tempting to speculate that, as is the case of mitochondrial TSPO and VDAC in peripheral-type benzodiazepine receptors, dimers of bacterial TSPO interact with a porin, namely OprF, and form hyper-structures in the membrane of *P. fluorescens*. The degree of homology of mitochondrial and bacterial TSPO is consistent with evolution from a common ancestor. In contrast, although VDAC and OprF are both porins, structural similarities between the two proteins are very low. Therefore, functional association between bacterial TSPO and OprF appears more as the result of evolutionary convergence. We should be then in this unique hyperstructure in the presence of a double and opposite evolutionary mechanism.

Although the natural ligand(s) of TSPO remains controversial, even in mammals, a first consequence of the present work is that it is highly probable that such molecules should be detected both by bacteria and eukaryotes and participate to inter-kingdom signaling. Communication between bacteria and eukaryotes using common molecules was only recently explored under the general identification of Microbial Endocrinology [37] but the consequences of such mechanisms are multiple. Indeed, since benzodiazepines are widely prescribed in medicine, these drugs are certainly exerting a continuous pressure on the physiology of the bacterial species of the human intestinal microflora. The resulting effect of these chronic and unsuspected interactions between benzodiazepines and the commensal microbiome remains to be investigated. On another side, this study suggests new hypothesis for the development of antibacterial molecules acting as virulence inhibitors. Indeed, by reducing the virulence of germs, PK 11195 or related molecules may allow the host to clear an infection without generating resistant forms of bacteria. This strategy is a new path to find alternative solutions to antibiotics as urgently requested by the World Heath Organization [38].

*(Use of PK11195 and derived molecules as inhibitors of virulence is covered by the French Patent No. 2 894 478 and is the property of ADIPpharm).*

## Materials and Methods

### Bacterial strains and culture conditions

*P. fluorescens* MF37 (biovar V) is a spontaneous mutant of the MF0 strain isolated from raw milk [15]. *P. fluorescens* clinical strains, MFY162 and MFN1032, are biovar I [14], whereas MFY63 and MFY70 are biovar II [39]. Clinical strain, MFN3597 is a *P. putida* biovar B [14]. For all strains, cultures were grown in Nutrient Broth (NB; Merck) medium (28°C, 180 rpm). Cultures were inoculated at an initial OD_580 nm_ of 0.08 and harvested in early stationary phase.

### Cloning and sequencing of bacterial *tspO* in MF37

The nucleotide sequence of MF37 bacterial *tspO* gene was deposited in the Gen-Bank database under accession number FJ185105. Primers 392-1 (CCGACCATGACCTTCTTCAT) and 392-1′ (GCCAGGTAAGGGAACAGGAT) were designed from the *P. fluorescens* SBW25 *tspO* genomic sequence (Sanger Institute http://www.sanger.ac.uk). Purified chromosomal DNA was PCR amplified using 35 cycles of denaturation (30 s, 94°C), hybridization (1 min, 60°C) and elongation (1 min, 72°C). The PCR fragment was cloned into the pMOSBlue vector, according to the supplier's protocol (pMOSBlue Blunt ended kit, Roche). Positive colonies were confirmed by PCR using the plasmidic universal primers, T7 and U19. Recombinant plasmids were extracted using the QIAprep Spin Miniprep (Qiagen). Plasmids were sequenced on both strands by Genomexpress (www.genome-express.com).

### In-silico analysis

The localization of TSPO and the predicted transmembrane structure were determined using the TopPred (http://mobyle.pasteur.fr/cgi-bin/MobylePortal) and PSORTb v2.0.4 (http://www.psort.org) programs [40]. The predicted structure of MF37 TSPO was compared to that of *R. sphaeroides* 2.4.1, recalculated from Yeliseev & Kaplan [12] and mitochondrial TSPO [13]. Existence of a peptidoglycan-binding signature (NXXLSXXRAXXVXXXL), based on the sequence of OmpA [27], was investigated. Homologies between the sequence of MF37 TSPO and those of other eubacteria were determined by searching the NCBI databank using the BLASTP program. Sequences were aligned using the multiple protein alignment algorithm, Clustal W [41], and analyzed using Philip version 3.5 (DNA parsimony program). The degree of statistical support for branches was determined with 1000 bootstrap replicates. The cladogram was visualized by Treeview 1.6.6. For analysis of the *tspO* genomic environment, flanking sequences in strains *P. fluorescens* Pf0-1 and *P. syringae pv.syringae* (B728a), *phaseolicola* (1448A), and *tomato* (DC3000) were retrieved from the Pseudomonas Genome Database (http://www.pseudomonas.com). For SBW25, 5,000 bp upstream and downstream of *tspO* were retrieved from the Sanger Institute (http://www.sanger.ac.uk) and translated into protein sequence (http://insilico.ehu.es). The hypothetical gene sequences were blasted against the prokaryotic NCBI database (http://www.ncbi.nlm.nih.gov) and for each one, the most likely homologue was included in the map of the genomic environment.

### PCR, Western blot and ESI MS-MS analysis

For PCR analysis, chromosomal bacterial DNA was extracted and subjected to PCR using primers designed from MF37 *tspO* to allow amplification of the last 363 bp ( Tav363F:AAA GGA TCC GGT ACG AAT CCC TGG TCA AA and Tav363R: AAA AAG CTT TCT TAT TCC GCA GGA TCG AG). PCR was performed using 30 cycles of denaturation (30 s, 94°C), hybridization (45 s, 60°C) and elongation (45 s, 72°C). For Western blot studies, proteins (40 µg), obtained from MF37 in early stationary phase, were separated by SDS-PAGE on a 15% acrylamide gel. Proteins were transferred for 30 min (490 mA) onto nitrocellulose membranes. Membranes were saturated for 1 h at 20°C in Tris-buffered saline (TBS: 20 mM Tris pH 7.5; 500 mM NaCl) containing 2% skim milk and 0.5% bovine serum albumin (BSA). Membranes were then incubated overnight at 4°C with rabbit anti-mouse mitochondrial TSPO [42] in blocking solution (1∶2,000). Membranes were rinsed twice with TBS containing 0.1% Tween 20 (TTBS) and incubated for 1.5 h at 20°C with alkaline phosphatase-conjugated goat anti-rabbit antibody (Biorad) in TTBS-1% BSA (1∶3,000). The colorimetric reactions were developed according to the instructions of the provider (BioRad). For electron spray ionization tandem mass spectrometry (ESI MS-MS), total bacterial proteins were extracted, as previously described [43], and an equivalent of 80 µg protein was separated by SDS-PAGE. The gel was cut vertically into two parts. One side was used for western blot analysis, while the other was stained with coomassie blue. The band containing TSPO-like immunoreactive protein was processed for ESI MS-MS analysis (UMR INRA1253, Rennes, France). The resulting peptide sequences were analyzed using “Findpeptool” software (http://www.expasy.org/tools/ findpept.html) to compare theoretical and experimental masses.

### Radioligand binding studies

Tritiated PK 11195 (^3^H-PK 11195; 73.6 Ci/mmole) was obtained from Perkin Elmer. Proteins were transferred onto nitrocellulose membranes and saturated for 1 h in the blocking solution already described. Membranes were rinsed twice in TBS and incubated for 30 min at 20°C in TBS containing 2.5 µCi/mL ^3^H-PK 11195. Membranes were then rinsed two times for 10 min in TBS and cut into 3 mm fragments. Each fragment was transferred to minivials containing 5 mL of scintillation liquid (Perkin-Elmer). For Scatchard studies, bacterial membranes were obtained by sonication on ice (four cycles of 30 s followed by microscopic control), adjusted to a final concentration of 60 µg membrane proteins/250 µl Tris-HCl buffer (50 mM, pH = 7.4), and incubated with ^3^H-PK 11195 at concentrations ranging from 0.6 to 59 nM. Mixtures were subjected to three steps of centrifugation (10 min, 10,500×*g*, 4°C) to remove membrane-bound tracer and rinsed with 0.5 mL ice-cold Tris-HCl buffer. Non-specific binding was determined by repeating the experiment in the presence of non-radioactive PK 11195 (30 µM).

### Biophysical studies

Due to its hydrophobicity, PK 11195 was dissolved in ethanol before mixing with culture medium. The final ethanol concentration was kept to 0.01%. All control experiments were performed in the presence of a same percentage of ethanol (0.01%). In normal oxygenation conditions, the culture medium corresponded to one-tenth of the volume of the culture vial volume, and one-half for reduced oxygenation conditions. Growth kinetics were determined by measuring OD_580 nm_. Swimming mobility was evaluated by puncture inoculation of plates containing NB supplemented with 0.3% agar. Swarming mobility tests were performed using an identical protocol, except that bacteria were deposited at the surface of NB supplemented with 0.5% agar. Bacterial adhesion to glass surfaces was studied using microscopic slides cleaned with ethanol and TFD4 detergent. Adhesion tests were performed by immersing slides for 2 h at 28°C in bacterial culture (10^8^ CFU/mL) and staining with acridine orange (0.01%, 20 min). For biofilm formation studies, bacteria were resuspended in NB at an OD_595 nm_ of 0.4. A constant volume of 100 µL of suspension was laid in a 96 well-plate and incubated at 28°C for 24 h in a water-saturated atmosphere. Each well was then rinsed and incubated in 0.1% crystal violet in water for 30 min. The excess of dye was removed and the crystal violet-stained biofilm was then dissolved with sodium dodecyl sulfate (1%), and the OD_595 nm_ of the extract was measured. Possible variations in the surface polarity of the bacteria were investigated using the microbial adhesion to solvent technique described by Bellon-Fontaine *et al.* [44] and hexadecane as hydrophobic solvent.

### Cell adhesion/invasion assays and cytotoxicity tests

Primary cultures of glial cells were obtained from newborn Wistar rats according to the recommendations of the French Ethics Committee (Agreements AGEXP27.01 and 27.06). The culture medium consisted of DMEM and Ham's medium (2∶1) supplemented with 10% fetal calf serum, 2 mM glutamine, 0.001% insulin, 5 mM HEPES, 0.3% glucose, and 1% antibiotic-antimycotic solution. Cells were allowed to grow for 12 days to reach confluence. Just before the infection test, bacteria harvested by centrifugation (6,000 rpm, 10 min) were resuspended in glial cell culture medium without antibiotics to a final concentration of 10^6^ CFU/mL. Effect of PK 11195-treatment on adhesive and invasive behaviors of bacteria was evaluated using the gentamicin survival assay [45]. The effect of PK 11195 on the cytotoxicity of *P. fluorescens* MF37 was determined through measurement of nitrite (NO_2_^−^) and lactate dehydrogenase (LDH) release by glial cells following a 24 h incubation in culture medium with bacteria. The use of these indirect methods for the study of apoptosis and necrosis has been previously validated [19]. Control studies were performed to verify the absence of nitrite or LDH production by bacteria. The concentration of nitrites in the incubation medium was determined using the Griess reaction. The concentration of LDH in the medium was determined using the Cytotox 96® enzymatic assay (Promega, France). LDH release was expressed as a percentage of the total amount of the enzyme generated through complete cellular lysis using Triton X-100 (9% [v/v]). The assays were sensitive enough to detect 0.5 µM NO_2_^−^ and a concentration of LDH equivalent to the lysis of 1% of the cell population. Values (mean±SEM) were calculated over a minimum of six independent experiments.
